# Effects of the Charge Density of Nanopapers Based on Carboxymethylated Cellulose Nanofibrils Investigated by Complementary Techniques

**DOI:** 10.1021/acsomega.4c00255

**Published:** 2024-04-25

**Authors:** Anna Maria Elert, Yong-Cin Chen, Glen J. Smales, Ievgeniia Topolniak, Heinz Sturm, Andreas Schönhals, Paulina Szymoniak

**Affiliations:** Bundesanstalt für Materialforschung und -prüfung (BAM), Unter den Eichen 87, Berlin 12205, Germany

## Abstract

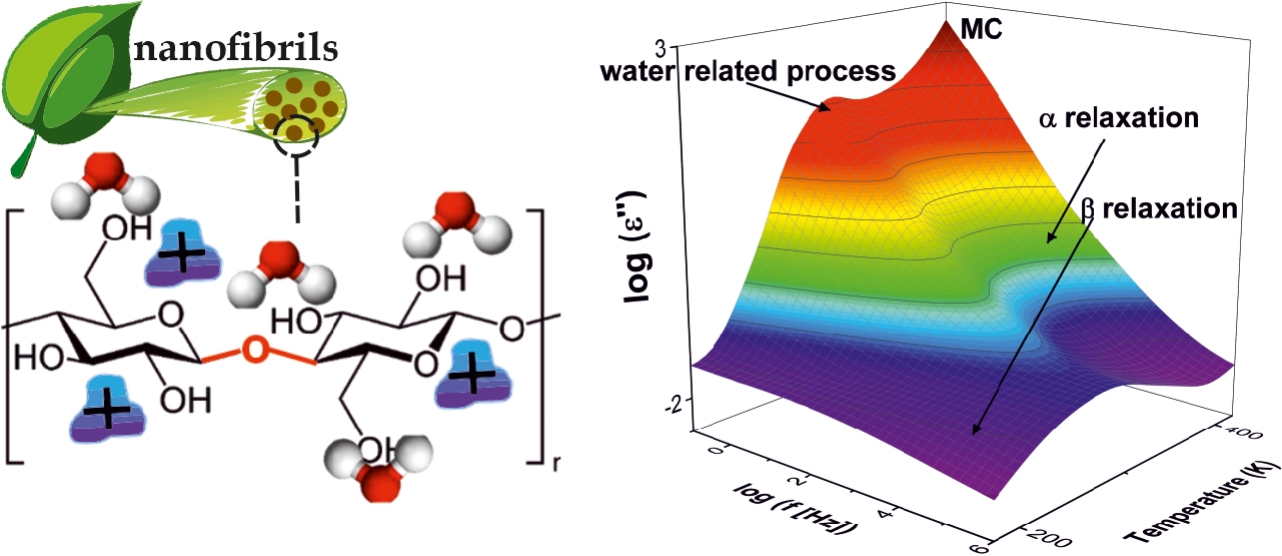

Cellulose nanofibrils (CNFs) with different charge densities were prepared and investigated by a combination of different complementary techniques sensitive to the structure and molecular dynamics of the system. The morphology of the materials was investigated by scanning electron microscopy (SEM) and X-ray scattering (SAXS/WAXS). The latter measurements were quantitatively analyzed yielding to molecular parameters in dependence of the charge density like the diameter of the fibrils, the distance between the fibrils, and the dimension of bundles of nanofibrils, including pores. The influence of water on the properties and the charge density is studied by thermogravimetric analysis (TGA), differential scanning calorimetry (DSC) and broadband dielectric spectroscopy. The TGA measurements reveal two mass loss processes. The one at lower temperatures was related to the loss of water, and the second process at higher temperatures was related to the chemical decomposition. The resulting char yield could be correlated to the distance between the microfibrils. The DSC investigation for hydrated CNFs revealed three glass transitions due to the cellulose segments surrounded by water molecules in different states. In the second heating scan, only one broad glass transition is observed. The dielectric spectra reveal two relaxation processes. At low temperatures or higher frequencies, the β-relaxation is observed, which is assigned to localized fluctuation of the glycosidic linkage. At higher temperatures and lower frequencies, the α-relaxation takes places. This relaxation is due to cooperative fluctuations in the cellulose segments. Both processes were quantitatively analyzed. The obtained parameters such as the relaxation rates were related to both the morphological data, the charge density, and the content of water for the first time.

## Introduction

Cellulose, a common form of polysaccharides found in the cell walls of plants, represents the most ubiquitous polymer source in the biosphere.^1^ From a chemical point of view, it is a linear polymer consisting of d-anhydroglucopyranose units (AGU) linked by β-(1,4) glycoside units. The AGU has three hydroxyl groups at the carbon positions C2, C3, and C6. Due to the presence of the −OH groups in the repeating unit, an inter- and intramolecular hydrogen-bonded network is formed, which leads to a highly ordered hierarchical spatial structure, ranging from the nano- to the microscale.^1,2^ Among all kinds of cellulose, cellulose nanofibrils (CNFs) receive an increasing attention.^2^ CNFs are bundles of stretched cellulose nanofibers consisting of crystalline and amorphous regions. They show superior properties, including high stiffness, large specific surface areas, a low weight, and a high biocompatibility. The intramolecular hydrogen bonds (interchain hydrogen bonds) provide the CNF with a high mechanical strength and make it insoluble in common solvents. The CNF organizes further into microfibrils. In wood, the microfibrils are embedded in a matrix of highly branched hemicellulose and lignin. As a renewable biopolymer with high availability in nature, cellulose shows potential in replacing mineral oil-based derivatives.

CNFs can be prepared by a top-down processing approach combining different physical and chemical treatments such as TEMPO-oxidation^3^ and carboxymethylation.^4^ These treatments are also commonly used to introduce anionic or cationic groups under different conditions like specific counterions with different ionic strengths and changing pH values or moisture content to control various properties such as the rheological behavior,^5^ swelling, and the formation of dry films.^6^

CNF-based materials are of great scientific and technological interest for a broad range of applications in insulation,^7^ fire-retardancy,^8^ packaging, energy storage,^9^ bioelectronics,^10^ and biomedicine.^11^ Further, CNFs can be considered as renewable building blocks of mechanically robust advanced functional materials. Due to their easy film-forming properties, they can be prepared in a large variety of forms such as transparent and flexible thin films, conductive nanopapers, lightweight porous materials, or strong filaments.^12^ The charged groups of CNFs can induce a polarization in the films which further enables the development of the next-generation sustainable electronic devices such as organic field-effect transistors (OFETs) or bioelectronics.^10,12^ For these applications, an in-depth understanding of the polarization behavior of films prepared from CNFs must be obtained. This can be achieved by investigating films with different surface charge densities, considering also different environmental conditions, such as humidity. The influence of moisture is one of the most critical factors affecting the electrical and thermal behavior of CNF-based materials.^13^ In general, water molecules act as a plasticizer in cellulose and influence different local molecular fluctuations and the segmental mobility.^14−16^

A suitable analytical tool to study the molecular mobility of a material is broadband dielectric spectroscopy (BDS).^17^ This method is highly sensitive to fluctuations of the dipole moments related to molecular groups and provides information about the structure–property relationship, taking dipoles as molecular probes for structure. Moreover, charge transport can also be studied by BDS. In the literature, several relaxation processes have been reported for polysaccharide cellulose derivatives and cellulose micro- and nanofibrils.^18−23^ An example for secondary relaxation processes is the so-called β-relaxation, observed at temperatures below the glass transition temperature *T*_g_. Secondary relaxations are ascribed to localized fluctuations within cellulose segments, or reorientation of the side groups. Form the literature, it is known that the localized relaxation processes as well as the glass transition might be strongly influenced by the presence of absorbed water as discussed above.^14−16^

Like other biobased materials, for cellulose, absorbed water can be categorized as “free” water, which has bulk-like properties of water, “freezing bound” water, which shows a freezing transition in a calorimetric experiment at temperatures different from that of bulk water, and “nonfreezing bound” water, which is not free and is not removable by drying the sample in vacuum at moderate temperatures.^24,25^

The influence of adsorbed water in its several states on the secondary relaxation of cellulose originates from various complex interactions of the water molecules with cellulose segments.^14^ For instance, absorbed water provides hydration states to the constituents of cellulose with a varying distribution of hydration levels. By studying cellulose nanofibrils of different origin, Lunev et al.^15^ concluded by dielectric measurements that at higher moisture contents, the morphology of the different raw materials strongly influences its ability to remain hydrated. It was also found that different states of water, such as free water (bulk-like) or a continuous water shell on the surface of nanofibril (saturated “solution” of CNF), can be present, which can also undergo a glass transition itself. The adsorbed water influences the local structure and the molecular mobility of cellulose segments but also affects the conductivity. Murphy et al.^26,27^ found that the electrical conductivity of cellulose at room temperature increases with increasing water content. This was explained by the formation of pathways of absorbed water molecules in cellulose, facilitating ion diffusion though the sample. Further studies conducted on humid cellulose powder were performed to develop models including percolation effects and hopping transport mechanism to discuss the observed results.^28,29^

To summarize, humidity control is crucial for the investigation of the molecular dynamics of cellulose. However, not only does the humidity influence the segmental dynamics of cellulose but also the charge density related to the degree of carboxymethylation can strongly affect it. This effect was investigated by Nessim et al.^30^ They investigated the influence of charged groups in the cellulose structure on molecular mobility. The study reports that the dielectric property of cellulose depends not only on the degree of substitution but also on the uniformity of the distribution of the charged groups.

Until now, there have been only a few investigations systematically discussing the influence of absorbed water and the charge density on the dielectric properties of cellulose. It is important to correlate both parameters since they mutually influence each other and can strongly affect the overall performance of the material. A further important factor, often not discussed in the literature, is the influence of a cyclical temperature treatment of the sample to the humidity content of the sample.

A first insight into the interaction between water and cellulose under isothermal dehydration was obtained by Zhao et al.^31^ Here, two distinct drying stages corresponding to water-dominated and cellulose-dominated states were found. However, the study was performed on cellulose powder, where the influence of a specific fiber morphology could not be investigated. The drying of CNF fibrils affects its performance, like the mechanical properties. It is expected that the electrical properties of the studied CNF could also be affected by dehydration, for instance, when the films are dried during one operation cycle and water cannot be reabsorbed for the next cycle.

Moreover, it is important to note that the sources, pretreatments, thermal history, and heat treatments of nanocellulose that influence the structure and composition on a molecular level are important parameters. Until now, most of the studies compared CNF from different sources with both different structural and chemical compositions including also different film preparation methodologies. Such an approach often led to problems in achieving an accurate conclusion for such a complex material.

Therefore, in this work, the studied samples are prepared from the same cellulose source (see materials and methods section), and a well-established carboxymethylation procedure^32^ was applied to vary the charge density, and in turn, control the chemical structure of the studied nanofibrils. CNF samples with a low charge (LC), medium charge (MC), and high charge (HC) density were prepared. Prior to dielectric studies, the CNF films were conditioned at a humidity of 75% to ensure a defined moisture content. To discuss the influence of the dehydration of the CNF films on its dielectric properties, the data from the first and second heating cycles were compared. During the second heating, most of the free and loosely bound freezing water of the CNF microstructure is removed from the film. Therefore, the molecular mobility observed during the second heating cycle is not affected by the presence of free or loosely bound freezing water anymore. To the best of our knowledge, such a comparison has not been presented before.

## Materials and Methods

### Preparation of the CNF Nanopapers

CNF gels with different charge densities were provided by RISE Bioeconomy (Stockholm, Sweden). In brief, the CNF gels were extracted from pulp (from Domsjö Fabriker AB) and carboxymethylated to obtain different charge densities prior to defibrillation. Carboxymethylation is a chemical modification of the hydroxyl groups of cellulose employing chloroacetic acid, leading to the replacement of some −OH by −CH_2_–COOH groups. The polar carboxyl groups endow cellulose with solubility and chemical reactivity. This might allow for a further introduction of polyelectrolyte multilayers with different charge densities. For this process, an earlier protocol proposed elsewhere^32^ can be applied. After the carboxymethylation procedure, the carboxyl groups were treated with different amounts of NaHCO_3_ to convert them to their sodium counterpart to obtain CNFs with different charge densities. For experimental details, see ref (32). The dissolved pulp had hemicellulose and lignin contents of 4 to 5 wt % and 0 to 1 wt %, respectively. Most residual lignin and hemicellulose were further removed during the carboxymethylation. The fibers were disintegrated using a high-pressure homogenizer several times to obtain CNF gels. The obtained CNF gels were diluted to 0.21–0.23 wt % through dispersion in Milli-Q water with an Ultra-Turrax device at 12 000 rpm for 10 min. The subsequent dispersions were then ultrasonically treated in a sonicator (10 min, 30% amplitude, 6 mm microtip probe) and then centrifuged at 4800 rpm for 1 h to remove aggregated pellets and to collect the supernatants as colloidally stable dispersion. The surface charge densities of the colloidally stable CNF dispersions were measured by polyelectrolyte titration (Stabino, Particle Metrix GmbH, Germany). The cationic polymer, poly(diallyldimethylammonium chloride) (PDADMAC) was used as titrant having a charge density of 0.307 μeq/mL. During the PDADMAC titration, the interface potential was measured as zeta potential representing the degree of electrostatic repulsion between CNF particles until the adsorption saturation at zero zeta potential. The total amount of PDADMAC solutions needed for each titration was then used to calculate the charge densities of the CNF dispersions. The surface charge density of the dispersions was determined to be 0.21 mmol/g (low charge density, LC), 0.42 mmol/g (medium charge density, MC), and 1.1 mmol/g (high charge density, HC). The dispersions of the CNFs in water were filtered through a Durapore filter (PVDF, hydrophilic, 0.65 μm) in a microfiltration assembly. Wet CNF films were formed after vacuum filtration and dried at 93 °C at a pressure of 95 kPa for 20 min using the drying section of a Rapid-Köthen sheet former (Paper Testing Instruments, Austria). The resulting film thicknesses were estimated to be between 23 and 26 μm for the nanopapers made from low and medium charge CNF dispersion and between 11 and 12 μm for the film prepared from the high charge CNF dispersion. Because several properties of the CNF, like the thermal ones, depend on the degree of crystallization, it should be mentioned that the carboxymethylation process did not change the degree of crystallization as evidenced below by X-ray scattering investigations.

Before the thermal and dielectric measurements, the samples were well conditioned under 75% humidity control.

### Differential Scanning Calorimetry (DSC)

DSC measurements were performed by a DSC 8500 instrument (PerkinElmer, USA) with heating and cooling rates of 10 K/min in the temperature range of −50 to 230 °C (223–503 K). Nitrogen was used as purge gas at a flow rate of 20 mL/min. Baseline corrections were conducted by measuring an empty pan under the same conditions as the sample. Indium was used as a calibration standard.

To estimate the thermal relaxation behavior of the CNFs, temperature-modulated DSC (TMDSC) measurements were carried out also employing the DSC 8500 device. For these measurements, the StepScan approach (Trademark PerkinElmer) was used. The covered temperature range was −50 °C to 180 °C (223–453 K). A heating rate of 60 K min^–1^ with a step height of 2 K was employed. The length of the isothermal steps resulted in frequencies between 6.66 × 10^–3^ and 3.33 × 10^–2^ Hz (corresponding to isothermal times 150 s down to 30 s). Again, nitrogen was used as the purge gas with a flow rate of 20 mL/min.

### Broadband Dielectric Spectroscopy (BDS)

The employed broadband dielectric spectrometer consisted of an active sample cell connected to a Novocontrol high-resolution ALPHA analyzer (Novocontrol, Montabaur, Germany). The dielectric properties of the sample characterized by the complex permittivity ε^*^(*f*) = ε′(*f*) – *i*ε″(*f*) (*f*: frequency; ε′ and ε″: real and imaginary part of the complex permittivity; *i* = ) were estimated in the frequency interval from 10^–1^ to 10^6^ Hz and in the temperature range from −100 to 200 °C (173 to 473 K). The temperature was controlled by a Quatro Novocontrol cryosystem with a temperature stability of 0.1 K. All samples were placed between two gold-plated electrodes (diameter = 10 mm) in a parallel plate geometry. The applied voltage was 1 V. For the thicknesses of the samples, see paragraph 1 (Preparation of the CNF Nanopapers). The impedance Z^*^(ω) was measured which is related to the complex dielectric function by
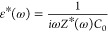
1

*C*_0_ is the geometric vacuum capacitance of the sample and *ω* is the radial frequency (ω = 2π*f*).

### X-Ray Scattering

X-ray scattering experiments covering the small and wide angles (SAXS/WAXS) were conducted by the MOUSE (Methodology Optimization for Ultrafine Structure Exploration) instrument.^33^ The MOUSE instrument is a highly customized Xeuss 2.0 instrument (Xenocs, Grenoble, France), where X-rays with Cu K_α_ and Mo K_α_ wavelengths (0.15 and 0.071 nm, respectively) are generated by microfocus X-ray tubes, and multilayer optics are used to parallelize and to monochromatize the beams. The beam was perpendicularly (90°) oriented to the surface of the sample, i.e., in an orthogonal view. The data were collected in vacuum by an Eiger 1 M detector (Dectris, Baden, Switzerland). The detector was placed at multiple distances between 52 and 2507 mm from the sample. The DAWN software package^34^ was used to process the measured data according to standardized procedures, also considering the propagation errors.^35^

### Scanning Electron Microscopy

To investigate the organization and packing of the CNF in the bulk, scanning electron microscopy (SEM) was employed. For these measurements, the films were immersed into liquid nitrogen, and a cross-section of the sample was obtained by breaking. The obtained cross-sections of the CNF film were analyzed with a Zeiss EVO MA 10 electron microscope (Carl Zeiss Microscopy GmbH, Jena, Germany) with a scanning electron microscope. An acceleration voltage of 10 kV and a secondary electron detector were employed for the measurements.

### Thermogravimetric Analysis (TGA)

TGA measurements were carried out using a Seiko TGA/DTA 220 instrument. The samples, with a weight of ca. 5 mg, were heated from 298 to 1273 K (25–1000 °C) with a heating rate of 10 K/min under a nitrogen atmosphere with a flow rate of 20 mL/min.

## Results and Discussion

### TGA Measurements

**Figure 1:**
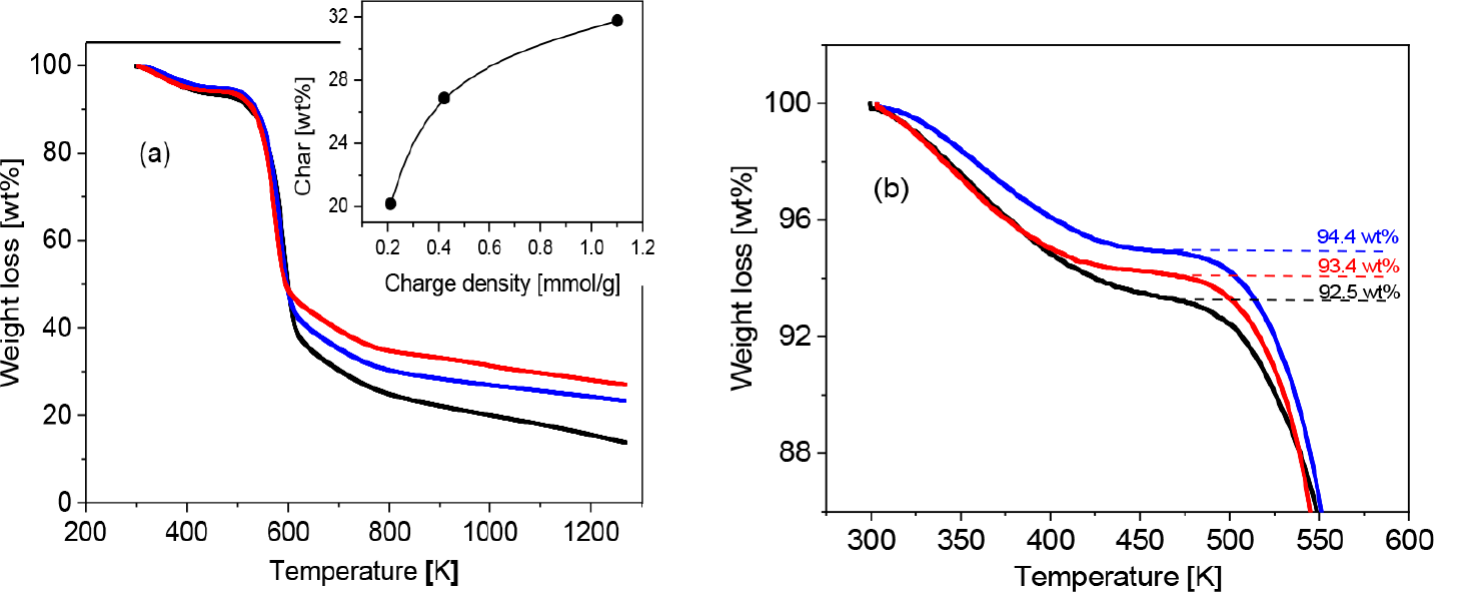
(a) Weight loss versus temperature for the different samples: black, LC; blue, MC; and red, HC. The inset gives the char in wt % at 1000 K versus charge density. The line is a guide to the eyes. (b) Weight loss versus temperature in the temperature range from 300 to 550 K for the different samples: black, LC; blue, MC; and red, HC.

The thermal stability of nanocellulose is one of the most important properties, determining the temperature range for its potential applications. In a TGA experiment, the weight of a sample is continuously monitored by a sensitive balance while the temperature is ramped with a defined rate. According to the purpose of the measurement, different sample atmospheres can be employed. Here, nitrogen was used. Figure 1a depicts the weight loss versus temperature for the investigated samples. For all samples, the weight loss is observed in two steps. In the temperature range from 298–523 K (25 to 250 °C) the weight loss is due to the evaporation of water from the samples (see Figure 1b). At first, it should be considered that during the conditioning of the samples at 75% humidity for each charge density, the same amount of water is absorbed. Figure 1b shows that for the sample with the lowest charge density, the weight loss due to water removal is the highest. This result suggests that for a lower charge density, fewer water molecules are tightly bounded to the cellulose segments and, as a result, more water molecules can desorb easily. However, the amount of lost water does not scale with the charge density (Figure 1b). Therefore, it is concluded that the charge density is not the only parameter which controls the desorption of water from the sample during temperature increase. The detailed morphology of the sample will also have most probably an influence on the water release.

However, it is not straightforward to assume that a conditioning of the samples at 75% humidity will result in the same amount of absorbed water for the different charge densities. As discussed for the water desorption process, the structure of the samples might also control the water absorption process. To investigate this hypothesis in more detail, experiments on samples that have been conditioned at different humidities should be carried out. Such experiments are in progress.

The strong weight loss at temperatures above 523 K to ca. 623 K is due to the chemical decomposition of the CNF. At temperatures above 623 K, the decomposition process slows down, reaching a “quasi”-plateau due to char formation. The char consists of carbonized material that cannot or only slowly further decompose. It should be noted that sodium is included in the char but is not the main part of it. In the inset of Figure 1a, the amount of char at 1000 K is plotted versus the charge density. The plot shows that the amount of char increases with the charge density. This result points to a denser structure of the nanofibrils formed for higher charge densities, which will be further discussed together with the X-ray scattering measurements below, including a correlation of the amount of char with measured structural parameters.

### Morphology

**Figure 2:**
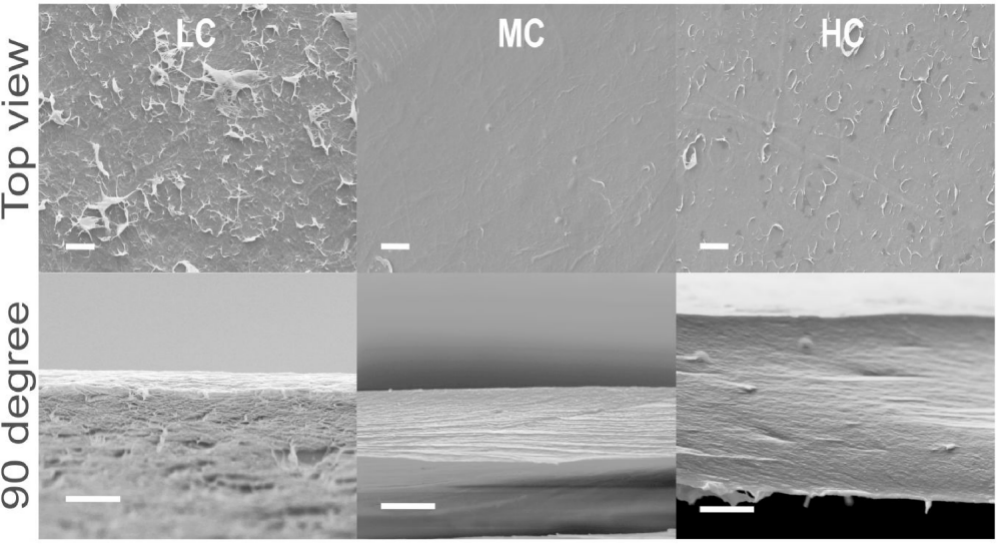
Scanning electron microscopy images of the CNF samples for different charge densities. The scale bars represent 5 μm.

Figure 2 shows electron microscopy images of the CNF samples for different charge densities. The images also shows that with increasing charge density, the structure of the samples becomes smoother and more compact.

**Figure 3:**
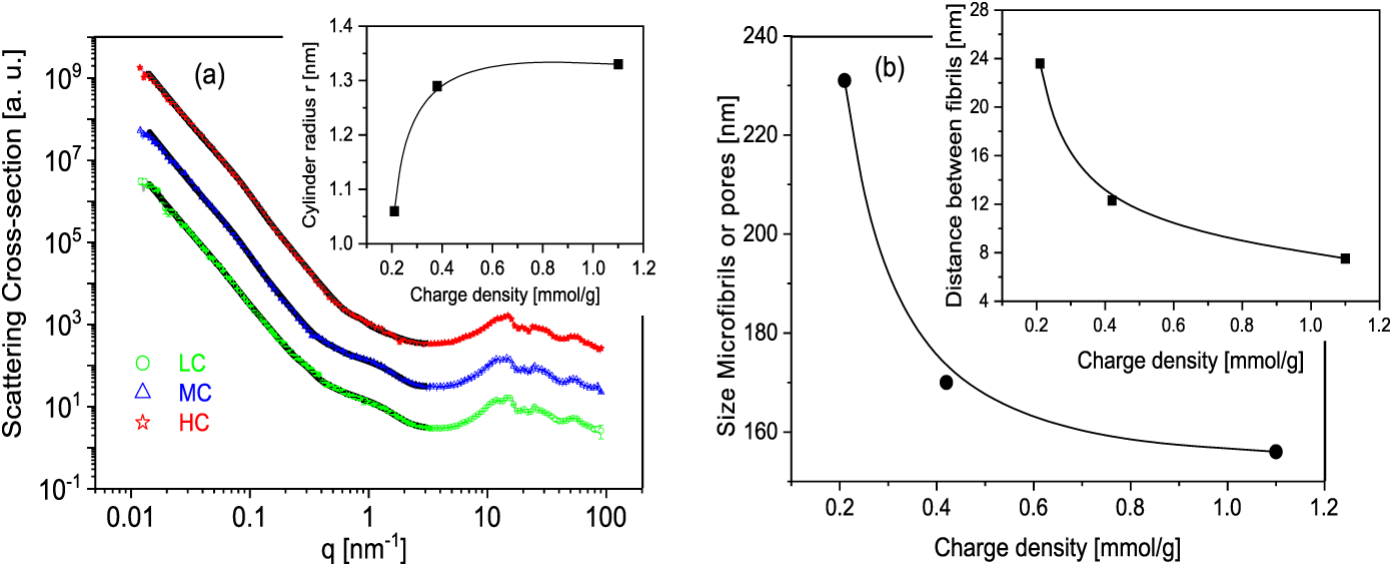
(a) X-ray pattern for the different samples: green, LC; blue, MC; and red, HC. Black lines are the fits of the WoodSAS model to the corresponding data. The curves are shifted along the *y*-scale for the sake of clarity. The inset gives the cylinder radius versus the charge density. The line is a guide for the eyes. (b) Size of the microfibrils and pores versus charge density. The inset depicts the distance between the fibrils versus the charge density. The line is a guide for the eyes.

For a more detailed analysis of the morphology of the samples, X-ray scattering measurements were carried out. Figure 3a depicts the X-ray pattern versus scattering vector *q* for the different charge densities. The WAXS region (*q* > 3 nm^–1^) shows Bragg peaks due to the crystalline structure of the nanocellulose fibers, which are almost identical for the different charge densities. This result means that the crystallinity of the CNF samples is not significantly influenced by the charge density. Besides the Bragg reflections, an amorphous halo is observed which is characteristic for the amorphous regions in the CNFs due to short-range correlation between carbon/carbon structures.^36^ Due to the high crystallinity of CNF and a broad peak distribution, as well as not very well-defined amorphous halo, the degree of crystallinity could not be estimated unambiguously.

In the SAXS region (*q* < 3 nm^–1^), an increase of the scattering intensity with decreasing *q* values is observed with some structural features around 1 nm^–1^ and 0.1 nm^–1^. These features are assigned to characteristic dimensions in the sample like the diameter of the nanofibrils, the dimension of bundles of nanofibrils, or sizes of holes (pores). The SAXS data is fitted in the *q*-vector range from 0.01 nm^–1^ to 3 nm^–1^ using the WoodSAS model^37−39^ which reads
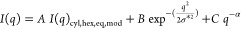
2

In short, *I*(*q*)_cyl,hex,eq,mod_ models the micofibrils as hexagonally packed cylinders. The term  describes bundles of nanofibrils including small holes whereas the part *q*^–α^ is used to describe larger pores. *A*, *B*, and *C* give the contributions of the different structural dimension of bundles to the whole scattering intensity. Figure 3a shows that the model describes the measured data reasonably well. The corresponding fit parameters are given in Table S1. It should be noted that the contrast for X-rays can be slightly different for the different charge densities, which can have an influence on the fitting parameters. As the fitting parameters obtained from the X-ray experiments correlate with results from other experiments, this effect should be minor. The inset of Figure 3a shows that the diameter of nanocellulose cylinders increases with increasing charge density. This result can be explained by considering electrostatic interactions, which increase with increasing charge density. It is interesting to note that the strongest structural changes took place when the charge density increased from a low to the medium value. With increasing charge density, the size of the microfibrils decreases (see Figure 3b), and similarly the distance between the microfibrils also decreases (see inset of Figure 3b). The latter result agrees with electron microscopy, which shows that samples become more compact with increasing charge density. Moreover, this result is in accordance with the dependence of the char formation on the charge density. In Figure S1, the distance between the fibrils obtained from X-ray scattering measurements is plotted versus the char in wt % at 1000 °C (1273 K) extracted from the TGA experiments. A linear correlation between both quantities is observed, which also supports this conclusion.

### DSC Measurements

**Figure 4:**
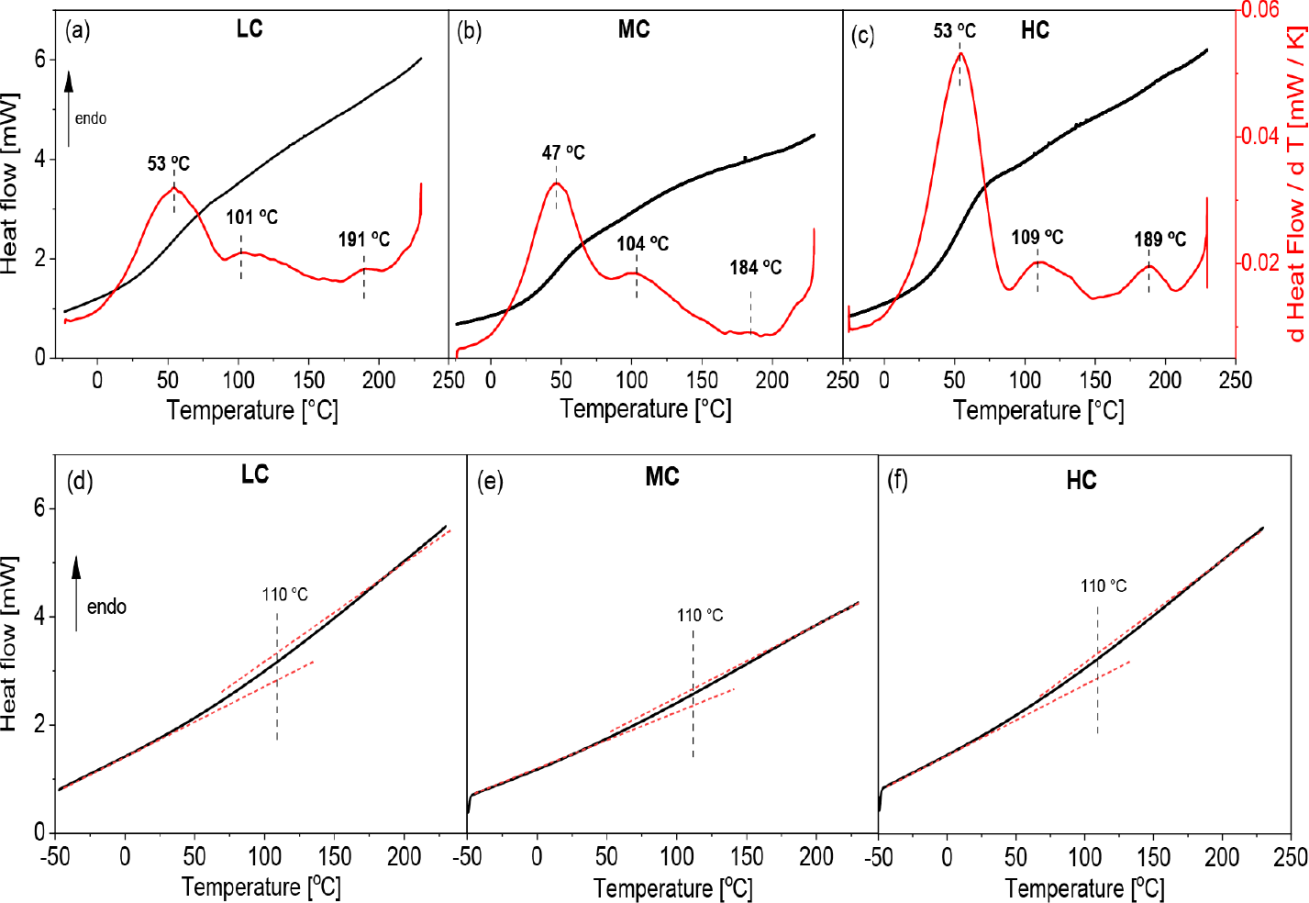
Heat flow (black line) and corresponding derivative (red line) versus temperature for CNF for the first heating run: (a) low charge density (LC), (b) medium charge density (MC), and (c) high charge density (HC). Heat flow versus temperature for CNF for the second heating run: (d) low charge density, (e) medium charge density, and (f) high charge density.

Figure 4a–c depicts the heat flow curves versus temperature for the CNF samples with different charge densities for the first heating run. All samples were well conditioned under 75% humidity before the measurement. The heat flow curves show a quite broad, step-like increase in the temperature range between −25 and 200 °C (248–473 K) which might indicate at least one glass transition region. Like for other semicrystalline polymers, the glass transition in CNFs is due to cooperative fluctuations in the amorphous regions of the samples. For further analysis, the heat flow curves were smoothed. Then, the first derivative with respect to the temperature was taken. From the peak maximum, a glass transition temperature *T*_g_ is extracted. For the first heating run for all charge densities, the derivative of the heat flow shows three peaks, where each peak is assigned to a separate glass transition. It should be noted that the three glass transitions could also be directly observed as weak steps in the heat flow data. Therefore, it is concluded that the derivative process creates no artifacts. One glass transition, *T*_g,1_, is observed at ca. 50 °C (323 K), a second one, *T*_g,2_, around 100 °C (373 K), and the third one, *T*_g,3_, around 190 °C (466 K). Each glass transition is related to the onset of the molecular mobility of cellulose segments surrounded by different amounts of water. Therefore, the glass transition at lowest temperatures is related to cellulose segments surrounded by free water, which introduces a strong plasticization to the segments leading to a relatively low *T*_g,1_ value. The second glass transition region at ca. 110 °C is assigned to cellulose segments surrounded by nonfreezing or tightly bound water. For this form of water, the plasticization effect is much weaker than that of free water. Basu et al.^40^ have reported the *T*_g_ of carboxymethylated cellulose to be at 79 °C (352 K) which is between the first and the second *T*_g_ reported here. As no detailed data analysis are given for these DSC measurements, the *T*_g_ value reported in ref.^40^ might be an average value of the first and second *T*_g_ values discussed here. A more careful consideration of Figure 4a–c reveals that the *T*_g_ value of the second glass transition (*T*_g,2_) shifts to higher temperatures with increasing charge density. In Figure S2, *T*_g,2_ is plotted versus charge density, with a linear correlation being observed in both cases. This result can be discussed considering that with increasing charge density, the interaction of the water molecules increases, leading to a slowing of segmental fluctuations and thus to an increase in *T*_g,2_. The third glass transition at ca. 190 °C (463 K) is related to dry cellulose segments. It was reported that the glass transition of dry cellulose is usually observed at a temperature higher than degradation temperature because of the highly cohesive nature of this biopolymer due to strong H-bonding between cellulose segments. The value of *T*_g,3_ for dry cellulose had been predicted to be between 220 and 250 °C (493 and 523 K).^41,42^

For the second heating run, no derivative analysis could be carried out, because the data have a larger scatter. Therefore, only the heat flow is considered (see Figure 4d–f). Nevertheless, it is observed that the first glass transition at about 50 °C (323 K) disappeared. During the first heating, especially at higher temperatures, water was evaporated from the sample. Therefore, the glass transition due to segments hydrated by free water cannot take place, because this structural feature does not exist anymore. Moreover, the second glass transition at ca. 100 °C (373 K), *T*_g,2_, is still observed as a step-like change in the heat flow. This observation supports the assignment of the second glass transition as to originate from cellulose segments surrounded by nonfreezing water, because this form of water cannot be removed in the first heating run. The last, high-temperature *T*_g,3_ at ca. 190 °C is not observed in the second heating run (see Figure 4d–f). One potential explanation is the broadening of the intermediate glass transition, which might overlay low-intensity *T*_g_ related to dry cellulose. The broadening and a slight increase in the value of the intermediate *T*_g_ which originates from cellulose segments surrounded by nonfreezing water might be due to a higher number of segments forming hydrogen bonds or other noncovalent interaction with the bound water and/or other nanocellulose segments. Upon heating, the free water is removed, and the segments which were plasticized in the as-prepared state interact with other entities of the system, leading to an increase of *T*_g_. In addition, the value of this glass transition temperature seems to be independent of the charge density. This might indicate that the amount of tightly bounded water is similar for all the samples. Nevertheless, the scattering of the data for the derivative of the heat flow for the second heating is large, limiting the possibility of detailed evaluation of the glass transition temperature value.

### Broadband Dielectric Spectroscopy

**Figure 5:**
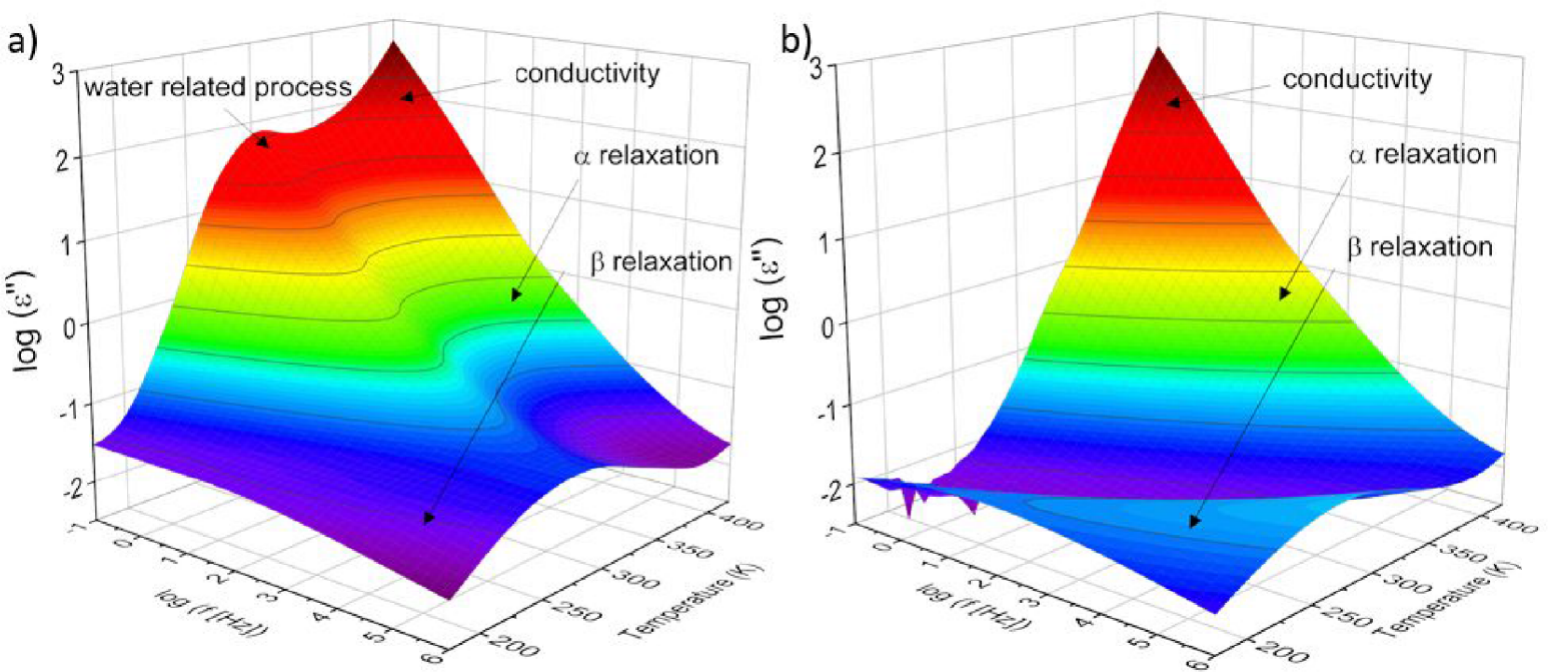
Dielectric loss versus frequency and temperature for the CNF sample with the medium charge density: a) first heating cycle and b) second heating cycle.

Dielectric spectroscopy is sensitive to the fluctuations of molecular dipoles and the drift motion of charge carriers. Figure 5 gives the dielectric loss versus frequency and temperature in 3D representations for the CNF with a medium charge density for the first heating (Figure 5a) and for the second heating cycle (Figure 5b). Several dielectrically active processes are observed in the dielectric spectra as peaks. A β-relaxation is observed at lowest temperatures or higher frequencies for the first and second heating cycles.

Further, for the first heating cycle, with increasing temperature, the β-relaxation is followed by a process in which the frequency position seems to be independent of temperature. Its intensity decreases strongly with increasing frequency. Such behavior is unusual for a relaxation process. Therefore, this process cannot be assigned as that. A similar process was observed for cellulose by Lacabanne et al.,^19^ as well as for a nanocomposite based on polypropylene and a layered double hydroxide by one of the authors here.^43^ Pissis et al.^44^ have related such a process to percolation effects. In a more systematic study on water confined to nanoporous glasses by Feldman et al., this process was assigned to an electric percolation through interconnected pores.^45−47^ By an analysis of the dielectric spectra in a quantitative way, information was obtained about the fractal nature of the pores as well as the porosity. The process observed here is also related to water because it disappears in the second heating cycle (compare Figure 5a,b). Due to the microfibrillar structure of the CNF samples, the systems contain a larger amount of interfacial area related to pores evidenced by the X-ray scattering data. Therefore, the assignment of this process to electrical percolation supported by water may also apply.

In addition to the already discussed processes, for the first heating cycle, the α-relaxation at higher temperatures is observed, but this process is partly hidden by the water-related process and the conductivity (see Figure 5a). The α-relaxation is more pronounced in the second heating cycle (Figure 5b) due to disappearance of the water-related process observed at lower temperatures. At even higher temperatures, a conductivity contribution to the dielectric loss is observed characterized by a strong increase of ε″ with decreasing frequency. In the following, the influence of water and the charge density on the different processes will be discussed.

#### Influence of Water and the Charge Density on the β-Relaxation

**Figure 6:**
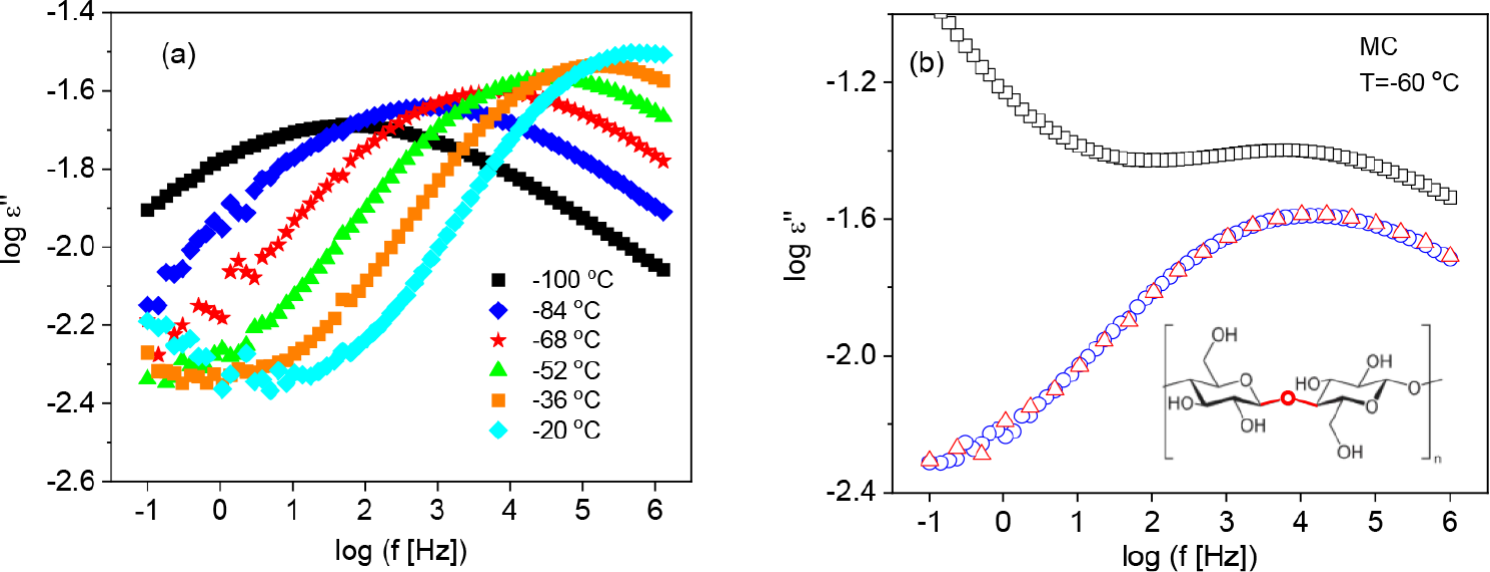
(a) Dielectric loss versus frequency for the indicated temperatures for the second heating cycle for the CNF film with the medium charge density. (b) Dielectric loss spectra at *T* = −60 °C (213 K) for the CNF film with the medium charge density for the first heating (black square), first cooling (blue circle), and second heating (red triangle; the number of available data points have been diluted). The inset gives the repeating unit of cellulose.

As discussed above, at lowest temperatures or highest frequencies, a broad β-relaxation process is observed. As an example, Figure 6a depicts the dielectric loss versus frequency at different temperatures for the CNF film with the medium charge density.

There is agreement in the literature that the β-relaxation is due to localized fluctuations. These could be fluctuations of side groups or localized fluctuations of the main chain. Here, the β-relaxation is assigned to the localized fluctuation of the main chain. As origin, molecular reorientations of glycosidic linkage can be discussed (see inset Figure 6b).^25^ However, there are also other arguments available in the literature about the interpretation of the β-relaxation of CNF including fluctuations of hydroxyl group,^40^ orientation of bound water molecules,^40^ and boat-chair interconversion of the pyranose ring.^24^ Nevertheless, fluctuations of the glycosidic bond are the most likely assignment because of its high dipole moment and the high intensity of the β-relaxation process.

Cellulose is a hydrophilic material and can easily absorb and bind water, as discussed in detail above. The water molecules bonded to cellulose can lead to additional contributions to the dipole moment and affect the dynamics of local fluctuations or cause additional localization to the relaxation processes.^14−16^ This is shown in Figure 6b where the dielectric loss is plotted for the sample with the medium charge density at −60 °C (213 K) for the different temperature cycles. For the first heating cycle compared to the first cooling and the second heating cycles, additional contributions to the dielectric loss are observed. First, it might be argued that they originate from a further underlying relaxation process. Such a process is associated in the literature with adsorbed water.^18^ Considering the decrease in the intensity of the process after the first heating cycle, this interpretation is supported by the experiments carried out here. During the first heating cycle, free water evaporates from the sample because during the measurement, the temperature is controlled by a dry nitrogen stream through the sample cell. Second, it can be argued that the additional contributions to the β-relaxation are due to the “water-related” process which is observed at higher temperatures (see Figure 5a). This process almost disappears in the second heating cycle, and therefore, its overlap with the β-relaxation is no longer observed. This becomes clear from Figure S3 where the dielectric loss is plotted versus temperature. For the second heating cycle, the β-relaxation becomes more visible as a peak. In the moment, it cannot be discriminated between the two possibilities, and further experiments are necessary.

The data for the β-relaxation were analyzed by fitting the empirical model function of Havriliak/Negami (HN-function) to the dielectric loss data.^48^ The HN-function reads
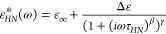
3where Δε denotes the dielectric strength. The shape parameters β and γ (0 < β; βγ ≤ 1) model the symmetric as well as the asymmetric broadening of the HN-function with respect to the Debye relaxation. τ_HN_ is a relaxation time related to the frequency of the maximal dielectric loss *f*_p_ (relaxation rate). Conductivity related contributions are treated in a conventional way by adding  to the loss part of the HN-function. Here, σ_0_ is related to the DC conductivity and *s* is a parameter describing nonohmic effects in the conductivity (0 < *s* < = 1). To remove the number of free fit parameters, γ is fixed to 1 (symmetrical peak). From the fits of the HN-function to data, the relaxation rate and the dielectric strength are obtained and further discussed below. An example of the fits is depicted in Figure S4. From the fits, the relaxation rate at maximal dielectric loss *f*_p_ is estimated by
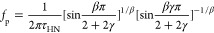
4

**Figure 7:**
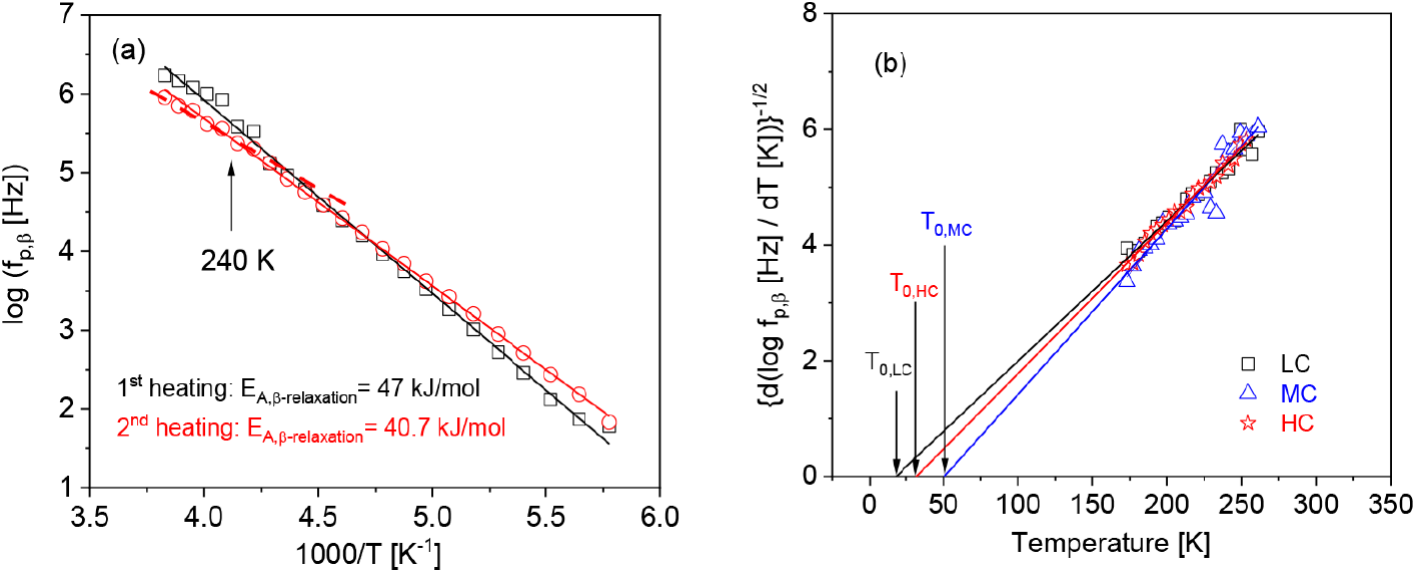
(a) Relaxation map for the β-relaxation for the CNF sample with medium charge density: black squares, first heating; red circles, second heating. Lines are fits of the Arrhenius equation to the corresponding data. The dashed red line is a fit of the Arrhenius equation to the data at the highest temperature until 240 K. (b) (d log *f*_p_/d*T*)^−1/2^ versus temperature for the samples with the different charge densities for the second heating cycle: black squares, LC; blue triangles, MC; and red asterisks, HC. Lines are fits of eq b6b to the corresponding data. The error of *T*_0_ is estimated from the linear fits and is ±5 K.

Figure 7a depicts the relaxation rate versus inversus temperature (relaxation map) for the β-relaxation for the CNF film with the medium charge density for the first and second heating runs. The data seem to be linear when plotted versus 1/*T* and can be approximated by the Arrhenius equation which reads
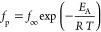
5a

Here, *E*_A_ is an (apparent) activation energy, *f*_∞_ is a prefactor corresponding to the relaxation rate at infinite temperatures, *T* symbolizes the temperature, and *R* is the general gas constant. For the first heating, an activation energy of 47 kJ/mol is estimated. For the second heating, the activation energy decreases to 40 kJ/mol, which is a non-negligible change. In the first heating cycle, the water molecules present in the system form hydrogen bonds with the −OH groups of cellulose segments, leading to a hydrogen-bonded network. These hydrogen bonds will restrict the localized fluctuations of the cellulose segments, yielding a higher activation energy to enable it. Due to the hydrogen-bonded network, the water molecules will participate in the β-relaxation at least for the first heating run. As a result of free water removal during first heating cycle, water molecules will no longer bond with the hydroxyl groups and the hydrogen-bonded network is destructed. The constrains for the localized fluctuation are removed easing the localized fluctuations responsible for the β-relaxation and lowering the corresponding activation energy.

The relaxation rates of the β-relaxation seem to not depend on the charge density. When plotted in the relaxation map for the different charge densities, they are collapsing more or less into one chart (see Figure S5).

The temperature dependence of relaxation rates can be analyzed in more detail by a derivative approach. This approach is sensitive to the functional dependence of *f*_p_(*T*) irrespective of the prefactor. For the Arrhenius equation, it holds
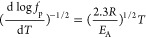
5b

This means, for a truly activated process, where the temperature dependence follows the Arrhenius equation, in the derivative representation, the data should follow a straight line going through the point of origin. In Figure 7b, the data for the β-relaxation for the samples with different charge densities are depicted in the derivative representation. The data follows a linear dependence, but this dependence cannot be extrapolated to the point of origin. The extrapolation to (d log *f*_p_/d*T*)^−1/2^ = 0 leads to finite temperature values. Such a behavior is characteristic for Vogel/Fulcher/Tammann (VFT) equation,^49−51^ which is given by
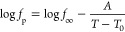
6a

Here, *A* is a fitting parameter and *f*_∞_ is a prefactor for the relaxation rate at infinite temperatures. *T*_0_ is the so-called Vogel or ideal glass transition temperature. Generally, a temperature dependence of the relaxation rates according to the VFT equation is considered as an indication for cooperative processes. The derivative of the VFT equation is given by
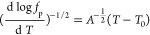
6b

Equation 6b also represents a straight line which gives for (d log *f*_p_/d*T*)^−1/2^ = 0 the Vogel temperature *T*_0_ which is found for conventional polymers to be 40 to 70 K below the glass transition temperature measured by DSC. The estimated *T*_0_ values for the β-relaxation are low but within the error range higher than 0 K. The VFT-like temperature dependence of the relaxation rates of the β-relaxation indicates that cooperative processes are involved in its molecular mechanism. Figure 7b shows that there is no correlation between *T*_0_ and the charge density. This result corresponds to the data obtained from the TGA measurements discussed above.

**Figure 8:**
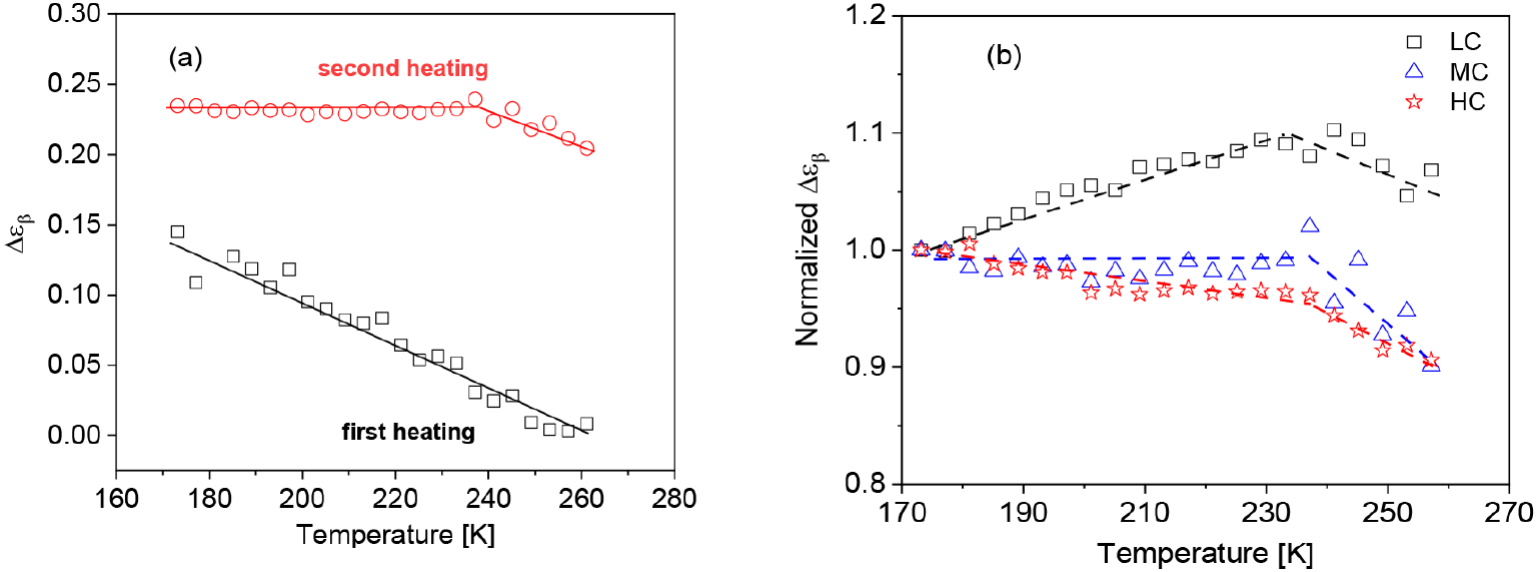
(a) Dielectric relaxation strength Δε of the β-relaxation versus temperature for the sample with medium charge density: black squares, first heating; red circles, second heating. Lines are guides to the eyes. (b) Dielectric strength of the β-relaxation normalized by its value at *T* = 173.2 K versus temperature for the second heating cycle for the samples with the different charge densities: black squares, LC; blue triangles, MC; and red asterisks, HC. Lines are guides to the eyes.

The second quantity obtained from the HN fits is the dielectric relaxation strength Δε. Unfortunately, the investigated CNF samples have a surface with high roughness. Therefore, the absolute values of the complex dielectric function are subject to larger errors. The same is true for Δε. For that reason, the temperature dependence of Δε for the different samples can be compared directly but not their absolute values. Figure 8a gives Δε_β_ for the β-relaxation versus temperature for the CNF sample with medium charge density. It is known that for conventional polymers, the temperature dependence of the dielectric relaxation strength for the β-relaxation increases with increasing temperature. Figure 8a shows that for the CNF sample with the medium charge density, Δε decreases with increasing temperature for the first heating cycle. This behavior can be explained considering that water molecules with a high dipole moment involved in the β-relaxation are desorbed from the sample. This means that during the first heating process, the net dipole moment of the sample is decreased, leading to a decrease of Δε_β_ with increasing temperature. For the second heating cycle, Δε seems to be independent of temperature until ca. 240 K (−33 °C). For temperatures above 240 K, Δε starts to decrease slightly. It is interesting to note that the change found in the temperature dependence of Δε_β_ seems to correspond to a change in the temperature dependence of the relaxation rates, as discussed above (see Figure 7a). Probably if higher temperatures are reached in the second heating, further changes take place in the samples due to a further loss of water molecules which might be more tightly bonded.

As discussed above, in general, the dielectric strength of the β-relaxation increases with increasing temperature. This temperature dependence was discussed, if either the number density of fluctuating units taking part in the β-relaxation increases or their fluctuation angle changes.^52^ The different behaviors found here for the CNF samples point to the fact that, in addition to these origins, further effects will contribute to the molecular mechanism of the β-relaxation. To discuss this further, Figure 8b compares the temperature dependence of Δε_β_ normalized by its value at *T* = 173.15 K for the samples with the different charge densities. It is observed that the temperature dependence of Δε_β_ depends on the charge density. For the lowest charge density (LC), Δε_β_ increases with increasing temperature until ca. 240 K. For the sample with the lowest charge density, this is the expected temperature dependence for a β-relaxation process. For the sample with the medium charge density (MC), Δε_β_ seems to be independent of temperature until 240 K, whereas for the sample with the highest charge density (HC), Δε_β_ slightly decreases with increasing temperature until 240 K. Like for the LC sample, for the samples with higher charge density, Δε_β_ decreases with increasing temperature for temperatures higher than 240 K. The number of sulfonated carboxyl groups increases with the charge density compared to the amount of hydroxyl groups. At first glance, one could argue that the ratio between the carboxyl and hydroxyl groups will cause the unconventional temperature dependence of Δε_β_ for the CNF samples. But if this is true, also other polymers having carboxylic groups should display a similar behavior, which is not observed. Remarkably, for all charge densities, a change in the temperature dependence of Δε_β_ takes place between 230 and 240 K, which is also not observed for conventional polymers. The difference between the system considered here and conventional polymers is that the CNF samples contain water. As the free water is evaporated during the first heating cycle, the remaining water molecules are confined and/or adsorbed on the cellulose nanofibers. In the literature, there is a long-standing discussion that nanoconfined water or ice undergoes a structural change (see for instance refs.^53−55^). A similar behavior was discussed by Lunev et al. for nanofibrilled cellulose.^15^ When it is assumed that the water nanoconfined in the CNF considered here also undergoes a structural change, it will also translate to localized fluctuations of the cellulose segments, because water molecules are adsorbed onto them. This will cause a change in the temperature dependence of the dielectric strength of the β-relaxation. If the structural change of the nanoconfined water alters the temperature dependence of Δε_β_, this should also be reflected in the temperature dependence of the relaxation rates. Therefore, Figure 7a is reconsidered. Indeed, the data at highest temperatures down to *T* = 240 K seem to have a different temperature dependence with a lower activation energy than the relaxation rates at lower temperatures. This can be considered as a further indication that the nanoconfined water in CNF undergoes a structural change in the temperature range between 220 and 240 K.

As was discussed in detail above, from the X-ray scattering experiments, the distance of the nanofibrils decreases with increasing charge density, whereas the diameter of the nanofibrils increases. These structural changes within the fibril will alter the molecular organization under which the fluctuations responsible for the β-relaxation will take place. In the ongoing work, this will be further investigated by temperature-dependent X-ray measurements.

#### Influence of Water and the Charge Density on the α-Relaxation

**Figure 9:**
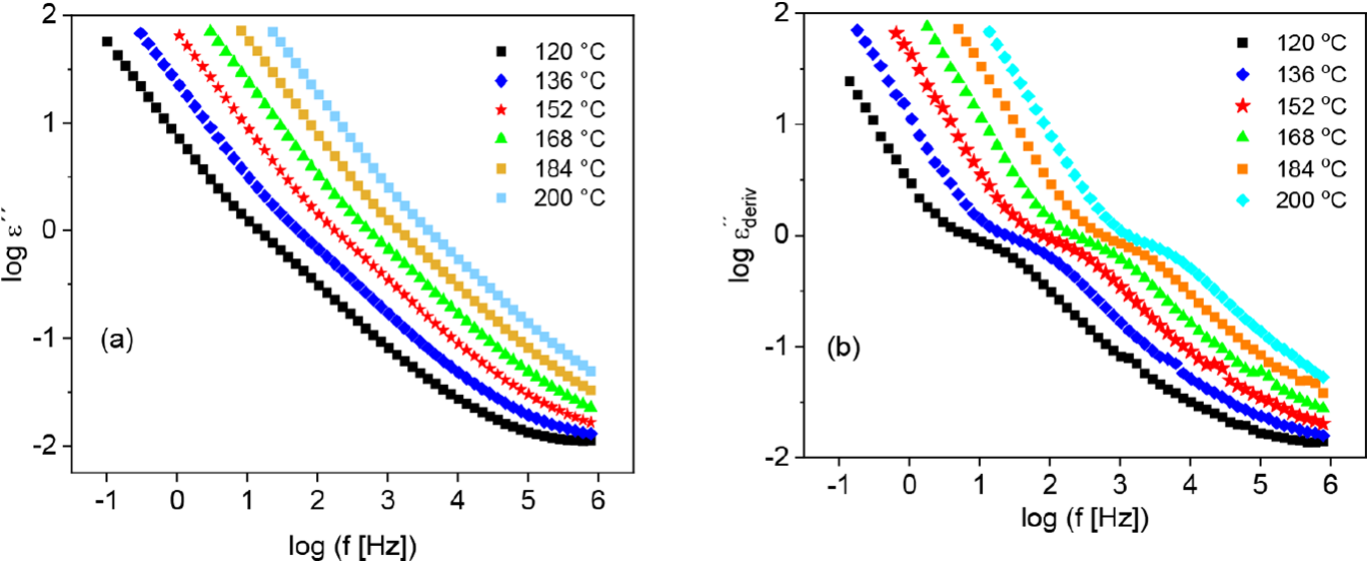
(a) Dielectric loss versus frequency in the temperature range of the α-relaxation for the sample with medium charge density at the indicated temperatures. (b) ε″_deriv_ versus frequency for the data given in part a of the figure.

There is not much information available in the literature about α-relaxation of cellulose because usually this process is not well visible in the frequency or temperature domain as it is covered by conductivity contributions and/or polarization effects like Maxwell/Wagner/Sillars or electrode polarization. This is demonstrated in Figure 9a where the dielectric loss is depicted versus frequency at different temperatures for the sample with the medium charge density in the temperature range for the α-relaxation. Some structure in the frequency dependence of the dielectric loss is observed, but no clear peak indicating the α-relaxation is visible. The problem of the overlaying conductivity to the relaxation process can be resolved by a derivative technique.^56^ In that approach, the real part of the complex dielectric function is differentiated with respect to frequency, as given by
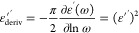
7

Because for a pure Ohmic conduction, the real part of the complex dielectric function is independent of frequency, Ohmic contributions to the conductivity are removed by that approach. For the Debye function, this procedure results in a peak for ε″_deriv_ which due to square in eq 7 is narrower than the peak in the dielectric loss itself.

In Figure 9b, this method is applied to the data given in Figure 9a. A peak is observed in the derivative representation which shifts to higher frequencies with increasing temperature, as expected. The behavior indicates a presence of a relaxation process, the α-relaxation. The increase in ε″_deriv_ with decreasing frequency observed for lower frequencies is due to polarization effects such as Maxwell/Wagner/Sillars and/or electrode polarization.

The frequency dependence of ε″_deriv_ was quantitatively analyzed by fitting the derivative of the real part of the HN function to the corresponding data. The derivative of the real part of the HN function reads
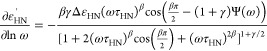
8awith
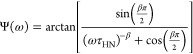
8b

Also, here, the parameter γ is fixed to 1. An example of the fit of the derivative of the HN-function to the data is given in Figure S6.

**Figure 10:**
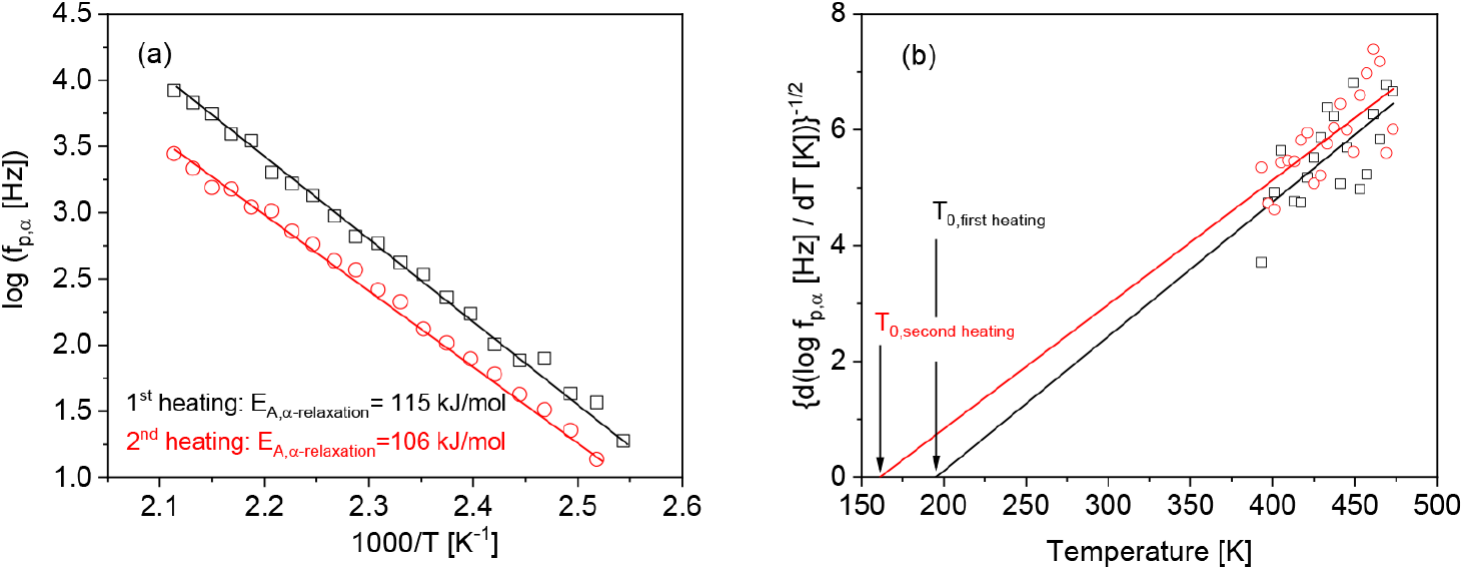
(a) Relaxation map for the α-relaxation for the sample with medium charge density (MC) for the first and second heating runs. Lines are fits of the Arrhenius equation to the corresponding data: black squares, first heating; red circles, second heating. (b) (d log *f*_p_/d*T*)^−1/2^ versus temperature for the sample with the medium charge density: black squares, first heating; red circles, second heating. Lines are linear regressions of the corresponding data.

Figure 10a depicts the relaxation rates of the α-relaxation versus the inverse temperature for the sample with the medium charge density for the first and second heating cycles. In general, one should expect a VFT-like temperature dependence of the relaxation rates for an α-relaxation. Figure 10a shows that this is not the case for the CNF samples investigated here. The data can be well approximated by the Arrhenius equation with apparent activation energies of 115 kJ/mol for the first heating and 106 kJ/mol for the second heating run. The difference in both activation energies is significant. Figure 10a shows further that the α-relaxation shifts to lower frequencies or higher temperatures for the second heating cycle. To prove whether the temperature dependence of the relaxation rates of the α-relaxation obeys an Arrhenius or a VFT behavior, the derivative approach (see eq 7) is again employed. The results are depicted in Figure 10b. Although the data have a larger scatter, this figure shows that the temperature dependence of the relaxation rates of the α-relaxation follows a VFT equation rather than an Arrhenius law. The appearance of the temperature dependence of the relaxation rates of the α-relaxation, such as the VFT dependence, points to a hindered glass transition. The molecular origin of the α-relaxation are the segmental fluctuations within the polysaccharide chain via the glucosidic bonds.^57^ As discussed, in the cellulose nanofibrils, the polysaccharide chains have highly ordered hierarchical structures, where bundles of cellulose nanofibers are stretched, forming crystalline and amorphous regions. Therefore, cellulose segments in the nanofibers can be considered as confined, leading to the observed hindered glass transition.^58−60^

The higher activation energy for the first heating cycle might be due to the water molecules which can form bridges or hydrogen bonds between the different segments which will restrict the molecular fluctuations responsible for the α-relaxation as discussed already for the β-relaxation.^25^

**Figure 11:**
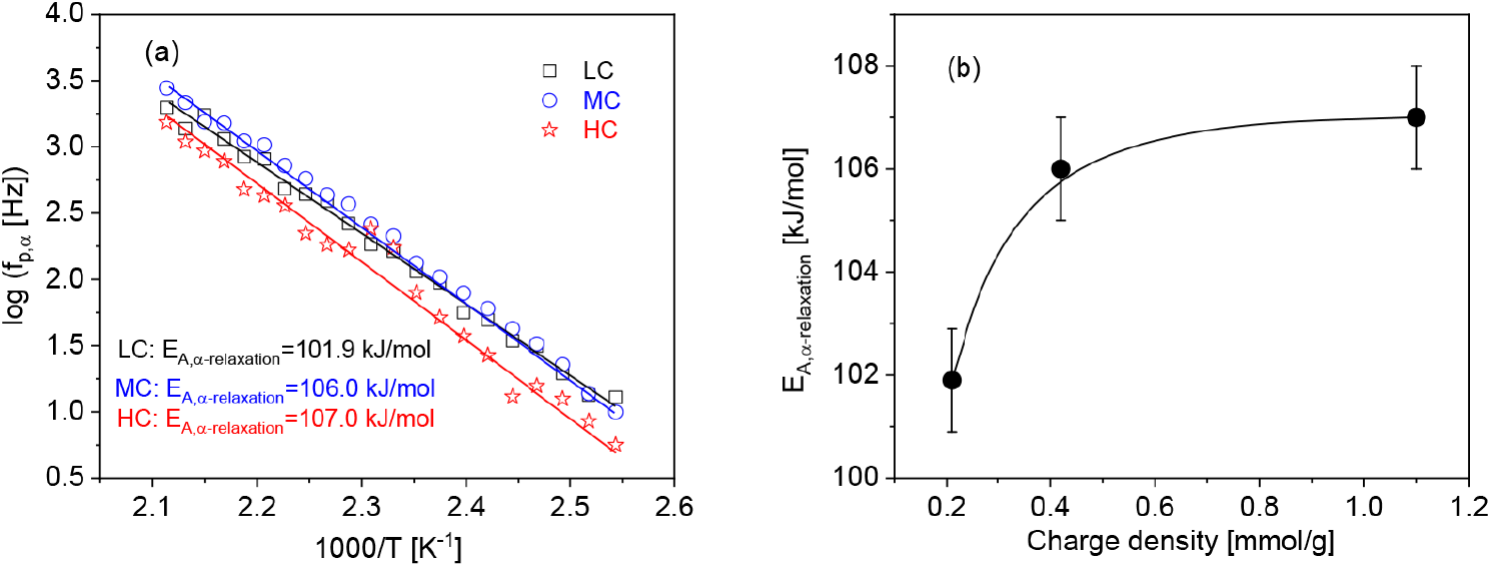
(a) Relaxation rates of the α-relaxation versus inverse temperature for the different charge densities for the second heating run: black squares, LC; blue triangles, MC; and red asterisks, HC. Lines are fits of the Arrhenius equation to the corresponding data. (b) Apparent activation energy versus the charge density. The line is a guide to the eyes.

Figure 11a compares the relaxation map for the α-relaxation for different charge densities. For all charge densities, the temperature of the relaxation rates of the α-relaxation rates can be approximated by an Arrhenius equation. The estimated apparent activation energies increase with increasing charge density, as shown in Figure 10b, where the strongest increase can be observed between the LC and the MC sample. Nevertheless, at a fixed temperature, the relaxation rates of the α-relaxation do not show a monotonous dependence on the charge density. This result agrees with the results obtained from the TGA measurements as well as for the β-relaxation.

**Figure 12:**
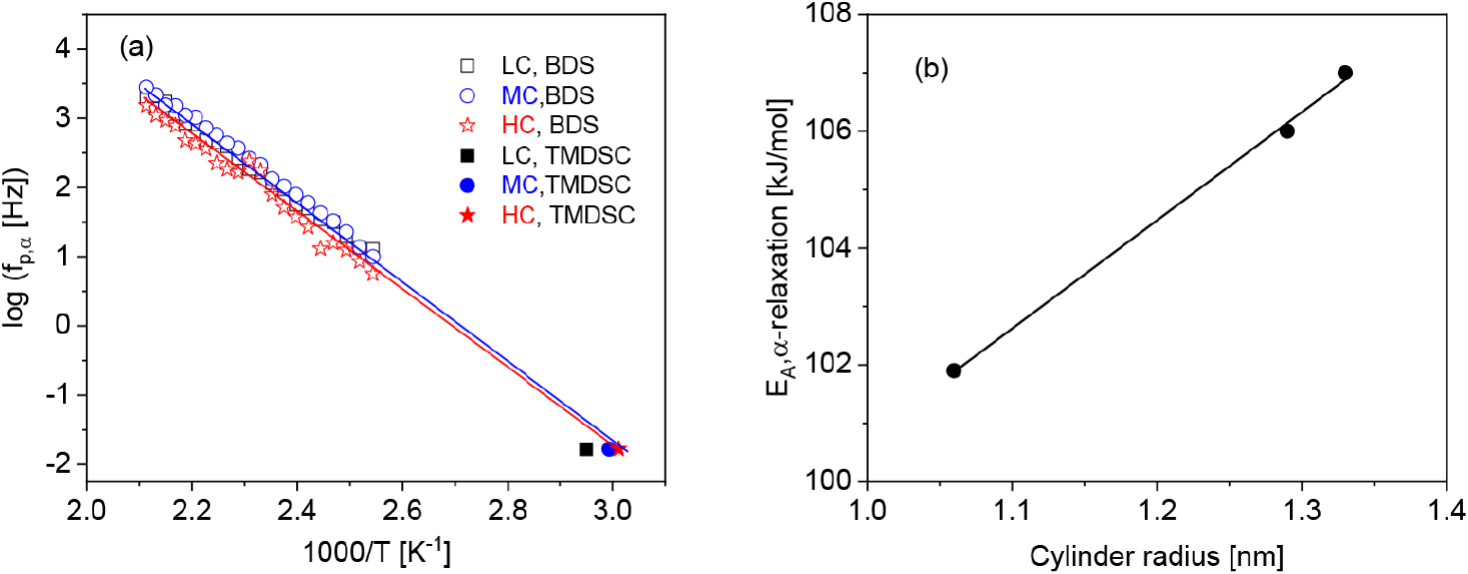
(a) Relaxation rate of the α-relaxation versus inverse temperature for the different charge densities for the second heating run: black squares, LC; blue triangles, MC; red asterisks, HC. Open symbols are measured by dielectric spectroscopy, andsolid symbols are data measured by TMDSC at a frequency of 1.67 × 10^–2^ Hz. Lines are guides to the eyes. Although the data seem to follow the Arrhenius equation, a more detailed analysis shows that they are better described by the VFT formula (see Figure 10b). (b) Apparent activation energy of the α-relaxation versus the cylinder radius. The line is a linear regression of the data.

To compare the dielectric α-relaxation with the calorimetric data, TMDSC measurements were carried out in the second heating run. An example for these measurements is givenin Figure S7. The temperature dependence of the relaxation rates of the α-relaxation can be well extrapolated to the glass transition temperatures measured by TMDSC (Figure 12a). This result proves that the data measured by BDS corresponds to a glass transition.

The α-relaxation takes place at the level of segments. Therefore, in Figure 12b, the activation energy of the α-relaxation is plotted versus the radius of the cylinders formed by the cellulose segments, obtained from X-ray scattering data. A linear correlation is observed between the diameter of the cellulose cylinder and the apparent activation energy of the α-relaxation. This result supports the assignment of this process to a hindered glass transition because with increasing diameter of the cylinders constraining effects to the fluctuation of the cellulose segments decrease. This effect will lead to a slight change of the Arrhenius-like dependence of the temperature dependence of the relaxation rates to a more VFT-like dependence, characterized by a higher apparent activation energy.

#### Conductivity and Polarization Effects

The complex conductivity σ^*^(ω) can be calculated from the complex dielectric function by
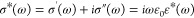
9

**Figure 13:**
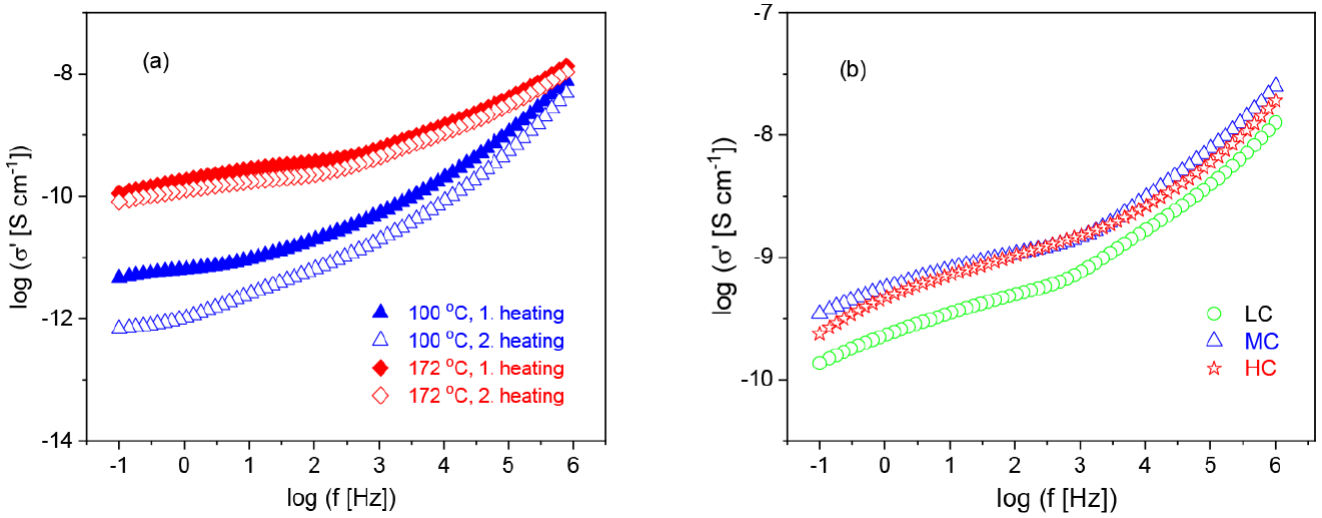
(a) Real part of the complex conductivity σ′ versus frequency for the indicated temperature cycles and temperatures for the sample with the medium charge density. (b) Real part of the complex conductivity σ′ in frequency domain 200 °C for different charge densities as indicated.

Here, σ′ and σ″ are the real and the imaginary parts of the complex conductivity, respectively, where ε_0_ is the permittivity of free space. Figure 13a depicts the real part of the complex conductivity versus frequency at two different temperatures for the first and second heating cycle. When the conductivity is due only to DC conductivity σ_DC_, σ′ should show a plateau at low frequencies where the plateau value gives σ_DC_. For none of the measured temperatures, a frequency independent plateau is observed. This means that frequency dependence of σ′ is not only due to conductivity but also to polarization effects like Maxwell/Wagner/Sillars (MWS) or electrode polarization. The MWS polarization is due to the blocking of charge carriers at internal interfaces. As known from the morphological investigations and from the literature, internal boundaries might be for instance the interface between amorphous and crystalline regions or the interface between different microfibrils. Therefore, a MWS polarization for CNF is most likely. The origin of electrode polarization is the blocking of charge carriers at the electrodes. In addition to the MWS polarization, the electrode polarization will contribute to σ′ too.

Figure 13a reveals that the conductivity measured during the first heating run is always higher than that measured during the second heating. This is likely due to the higher content of water in the CNF during the first heating. In the presence of water, a proton can be abstracted form the sulfonated carbonyl group.^35^ For a higher water content, more protons can be abstracted, leading to a higher conductivity. As the temperature is increased to 100 °C (373 K) and higher values are reached, the difference between the real parts of the complex conductivity in the first heating and the second heating decreases. This result supports the conclusion that water is responsible for the higher conductivity in the first heating run as water evaporates at higher temperatures, and this effect should vanish.

It should be noted that like the complex permittivity, the absolute values of the complex conductivity are subjected to larger errors because of the roughness of the sample surface. Nevertheless, in Figure 13b, σ′ is plotted versus frequency for the different charge densities at a temperature of 200 °C (473 K). The real part of the complex conductivity σ′ does not vary with the charge density. It might be discussed that the real part of the complex conductivity of the CNF with the medium and high charge density CNF is higher than that of the CNF with the low charge density. This might be due to more free charge carriers.

## Conclusion

Cellulose nanofibers with different charge densities were prepared using CNFs from the same source and investigated by a combination of complementary methods. Thermal analysis reveals two-stage decomposition processes for CNF, the first, where a mass loss step is related to the loss of water, and the second being a mass loss step where CNF starts to degrade at 210 °C. The results show different thermal stabilities with the higher charged CNF exhibiting a higher thermal stability compared to that with lower charge densities. This result is discussed in relation to samples with a higher charge density, forming a denser structure of nanofibrils than those with lower charge densities. This conclusion is supported by structural investigations using electron microscopy and X-ray scattering (SAXS/WAXS). DSC analysis of the CNF samples hydrated at 75% humidity show three glass transition temperatures at ca. 53 °C 101–110 °C, and 193 °C, corresponding to CNF segments surrounded by free water (1st *T*_g_), CNF segments in an environment with bounded water (2nd *T*_g_), and dry CNF segments (3rd *T*_g_). A change of the glass transition temperature with the charge density is detected only for medium wet CNF (2nd *T*_g_) which correlates linearly with the charge density.

BDS measurements show two relaxation processes: the β-relaxation (related to localized fluctuation) and the α-relaxation (related to cooperative segmental fluctuations). An additional process with peculiar properties related to water and the nanostructure of CNF is observed only for wet CNF and takes place at temperatures between the β-relaxation and the α-relaxation approximately at room temperature. The β-relaxation is assigned to localized fluctuations of the glycoside linkage in the monomeric unit. For the first heating run, where the sample contains free water, bridges are formed, which restricts the fluctuation responsible for the β-process expressed by a higher activation energy. From a detailed analysis of the temperature dependence of the relaxation rates and dielectric strengths, it is concluded that fluctuations of the β-relaxation involve some cooperative elements. The influence of charge density on the segmental motion in CNF was also investigated by BDS. No significant difference of the activation energy of the β-relaxation for the different charge density CNF is observed.

The α-relaxation related to segmental motions of the polysaccharide chain via the glucosidic bond of the CNF could be analyzed quantitatively by a derivative approach (conduction free loss). Surprisingly, the temperature dependence of the relaxation rates could be described by the Arrhenius equation with a high apparent activation energy due to a hindered nature of the glassy dynamics. For the second heating run, the apparent activation energy decreases. This is discussed by the loss of water. The water bridges between polymer segments also restrict the cooperative motion of CNF. For the second heating run, the free water is lost and the restriction to the segmental fluctuations is removed, leading to a decrease in the apparent activation energy. It is worth noting that the temperature dependence of the α-relaxation can be extrapolated to the data measured by TMDSC which indicates that both measurements probe the same process, the glass transition. The Arrhenius-like temperature dependence of the relaxation rates of the α-relaxation is discussed in the frame of a hindered glass transition. The findings presented here prove that the confinement of segmental motions has two origins. First, it arises from the ordered nature of the nanofibrils and second from the formation of H-bonds with water molecules. This argumentation is supported by a linear correlation of the apparent activation energy of the α-relaxation and the diameter of the microfibrils as well as the above-mentioned decrease of the apparent activation energy upon the second heating run. Nevertheless, the selected sample system is quite complex. Therefore, further investigations are planned. This might include FITR experiments but, more importantly, a variation of the humidity.

## Supporting Information Available

Fitting parameters X-ray scattering; distance between the fibrils versus the amount of char; *T*_g,2_ versus charge density; dielectric loss versus temperature for different heating cycles; examples for fitting of the HN-function to the data; relaxation map for the β-relaxation; example for the fit of the derivative of the real part of the HN-function to the conduction free loss; example for a TMDSC measurement (PDF)
