# Supplementary material for: Effects of the Charge Density of Nanopapers Based on Carboxymethylated Cellulose Nanofibrils Investigated by Complementary Techniques
Source: ACS Omega. 2024 Apr 25;9(18):20152–66. doi: 10.1021/acsomega.4c00255 (PMC11079888; doi:10.1021/acsomega.4c00255)
Supplement: Supplementary file 1 — ao4c00255_si_001.pdf [file ao4c00255_si_001.pdf]

Supporting Information

Effects of the charge density of nanopapers based on carboxymethylated cellulose nanofibrils investigated by complementary techniques.

Anna Maria Elert, Yong-Cin Chen[§], Glen J Smales, Ievgeniia Topolniak, Heinz Sturm, Andreas Schönhals, Paulina Szymoniak[*]

[1]Bundesanstalt für Materialforschung und -prüfung (BAM), Unter den Eichen 87, 12205 Berlin, Germany.

[*]Coresponding author: P. Szymoniak, Bundesanstalt für Materialforschung und -prüfung (BAM, Fachbereich 6.6), Unter den Eichen 87, 12205 Berlin, Germany; Tel. +49 30 / 81044799; Fax: +49 30 / 8104-74799; Email: Paulina.Szymoniak@bam.de

[§]Current address: MKS Instruments, Atotech Deutschland GmbH & Co. KG
Erasmusstraße 20, 10553 Berlin, Germany

Table S1: Parameters extracted from the X-ray scattering investigations using the WoodSAS model.

Sample	Cylinder radius [nm]	Distance between the fibrils [nm]	Size of the microfibrils bundles/pores [nm]
LC	1.06	23.6	231
MC	1.29	12.31	170
HC	1.33	7.53	156

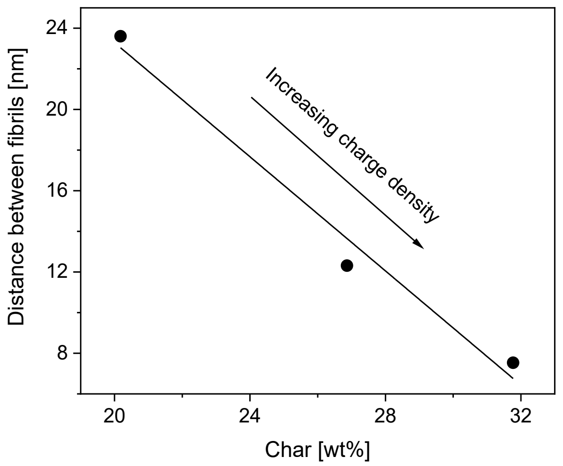

Figure S1: Distance between the fibrils versus the amount of char obtained from the TGA measurement at T=1273 K.

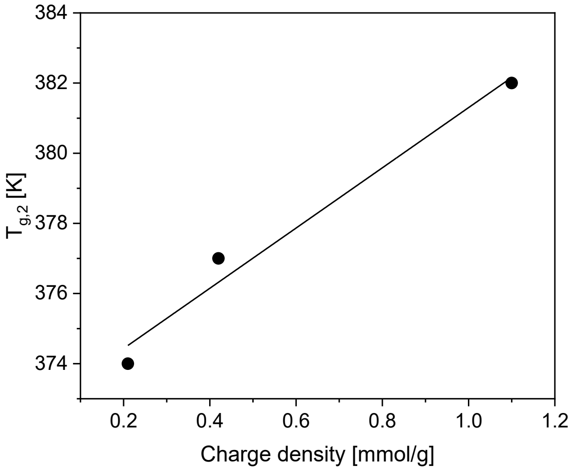

Figure S2: $T_{g,2}$ versus charge density. The line is a linear regression to the data.

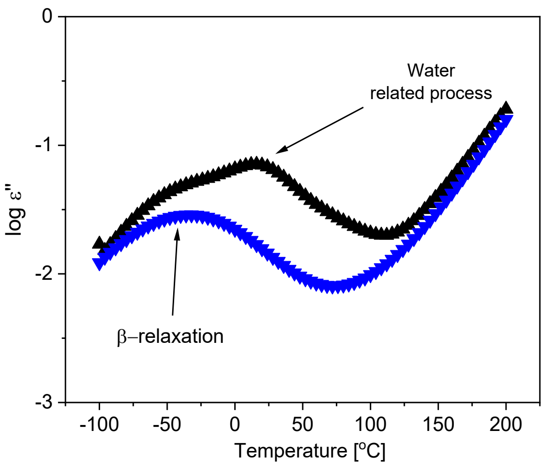

Figure S3: Dielectric loss versus temperature at a fixed frequency of 100 kHz: black triangles – first heating, blue down sited triangles – second heating.

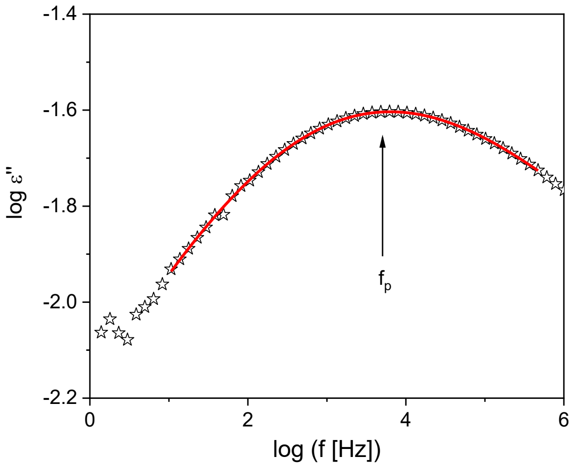

Figure S4: Example for the fit of the spectra of the β-relaxation for the CNF sample with the medium charge density at T=205 K.

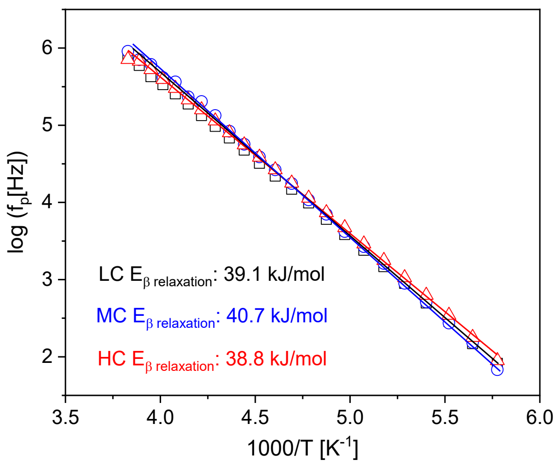

Figure S5: Relaxation map for the β-relaxation: black -LC, blue-MC and HC. The lines are fits of the Arrhenius equation to the corresponding data.

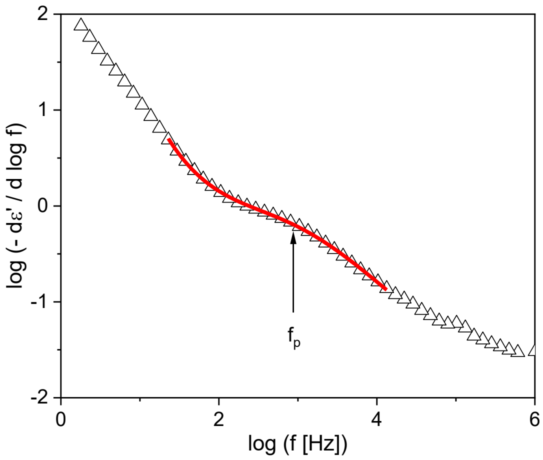

Figure S6: Example for the fit of the derivative of the real part of the HN-function to the conduction free loss for the sample with the medium charge density. The red line is the fit to the data.

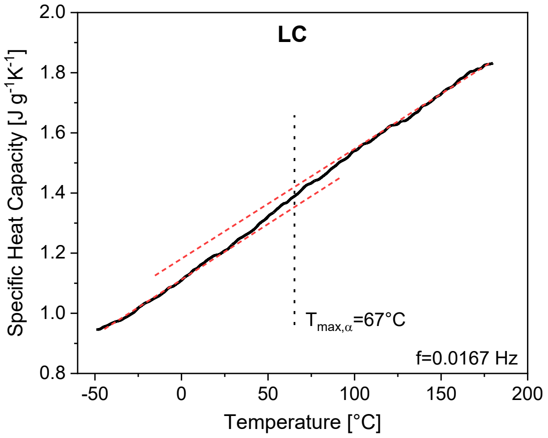

Figure S7: Example for a TMDSC measurement for the sample with the low charge density at f=0.0167 Hz.
